# Composites with high omnidirectional fracture toughness due to helical interlocking fasteners are found in gingko seed shells

**DOI:** 10.1093/nsr/nwad065

**Published:** 2023-03-28

**Authors:** Nicholas A Kotov

**Affiliations:** Department of Chemical Engineering, Biointerfaces Institute, Department of Materials Science and Engineering, University of Michigan, USA; Department of Aeronautics, Imperial College London, UK

Engineering of composites mimicking load-bearing materials from living organisms is essential for sustainable economic development and successful resolution of current and future bottlenecks of chemical, automotive, aviation, biomedical, and robotic technologies. As pointed out by G. Wiedemann [1] in 1853 and M.F. Ashby *et al.* [2] in 1995, the properties of traditional materials property are strongly correlated. Biomimetic nanocomposites, on the other hand, provide a combination of properties that are impossible to attain by other means making a tremendous difference to human lives. For example, biomimetic composites replicating the structure of seashell lining nacre known for its mechanical properties exceeding those of individual components multiple times [3], are already being utilized in various products starting from barrier coatings and car bumpers to flexible electronics and neuroprosthetic implants. Particularly successful are nacre-like composites from graphene oxide that can also produce conductive coatings [4]. Importantly, nanoscale components capable of self-organization offer a general platform for the engineering of biomimetic nanocomposites with multiple properties. One of the properties materials in nature are optimized for is fracture toughness [5], K_IC_, which is essential for virtually every application. High values of K_IC_ in nacre-like composites and other materials originate from the crack deflection, which has preferential direction due to strong anisotropy of nanoplatelets. However, materials with weakly anisotropic structure that can effectively resist omnidirectional propagation of cracks are equally needed. Even natural prototypes of such materials are hardly known.

**Figure 1.**
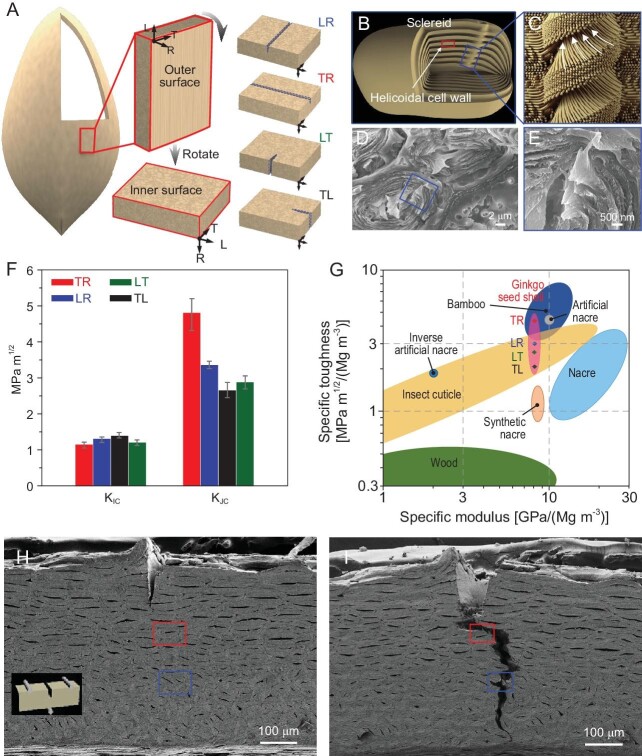
(A) Four directions of crack orientations. (B) Schematic illustrations of a sclereid with a pit embedded in its wall (B) and the intact pit shows a helical fastener structure (C). (D) Scanning electron microscopy (SEM) micrographs of fracture morphology shows a helical pit integrated into cell walls. (E) A higher magnification SEM image of a helicod. (F) K_IC_ and K_JC_ for the four crack orientations. (G) Comparison of the specific fracture toughness and specific modulus of the ginkgo seed shell in four crack orientations, along with other natural and artificial materials. (H and I) Fracture morphology of the single-edge notched sample with the crack orientation. Reproduced from Ref. [6].

Recently, a research team led by Professor Qunfeng Cheng at Beihang University, has discovered ginkgo seed shells display weak anisotropy of mechanical properties and have excellent resistance to omnidirectional crack propagation (Fig. 1A) [6]. They found that these biological composites are tightly packed by sclereids cells interconnected by helical fasteners (Fig. 1B–E). This design is quite ingenious because it combines interlocking microbridges found at the hard–soft interface in nacre [7] and helicoidal geometries known for efficient energy dissipation. Three-point bending test for four orientations of crack propagation displayed similar values of K_IC_ = 1.26 MPa m^1/2^, while cracks growing in four directions display the highest K_JC_ up to 4.82 MPa m^1/2^ (Fig. 1F). The *in-situ* electron microscopy imaging (Fig. 1H and I) demonstrated that the cracks tend to branch due to the interlocking of different microscale segments. Energy dissipation increases because sclereids unit can deflect the crack tip and bridging the emerging gaps. In the inner layer of the ginkgo shell, the cracks have a longer zigzag-shaped track (Fig. 1I), which makes the crack deflection even more efficient. Besides having the unusual property of omnidirectionality, the authors also found that the overall values of fracture toughness of ginkgo seed shell compare favorably with other natural [2] and man-made materials [8], such as abalone shells [9], insect skins, wood and bamboo (Fig. 1G).

This work of the Prof. Qunfeng Cheng made a novel contribution to the development of hierarchical biomimetic composites. The knowledge of the complex organization patterns, that makes possible high toughness, greatly facilitate their replication using abiotic materials, potentially by using self-assembly processes. Replication of the helical fasteners integrated with the composite to connect microscale blocks discovered in this article can be the next step in this study. The advances in the self-assembly of the chiral nanoparticles into helicoids with similar dimensions and pitch make it realistic [8]. Utilization of the metallic or semiconductive nanostructured components instead of cellulose-based insulating building blocks and dielectric blocks typical for plants will also make possible *in-situ* deformation and damage sensing that would be essential for all the technologies mentioned above.
